# Latent resting-state network dynamics in boys and girls with attention-deficit/hyperactivity disorder

**DOI:** 10.1371/journal.pone.0218891

**Published:** 2019-06-28

**Authors:** John E. Scofield, Jeffrey D. Johnson, Phillip K. Wood, David C. Geary

**Affiliations:** Department of Psychological Sciences, University of Missouri, Columbia, Missouri, United States of America; University of Udine, ITALY

## Abstract

Neuroimaging studies of subjects with ADHD typically show altered functional connectivity in prefrontal, striatal, and several temporal brain regions. While the majority of studies have focused on connectivity that is averaged over time, we investigated the temporal dynamics of brain network changes in resting-state *f*MRI. Using the ADHD-200 consortium, we characterized the time course of latent state changes using Hidden Markov Modeling, and compared state changes between boys and girls with ADHD along with typically developing controls. Sex differences were found in latent state switching, with boys dwelling longer in a given state than girls, and concurrently having fewer overall state transitions. These sex differences were found in children with ADHD and in typically developing controls. Children with ADHD were also found to be more variable in terms of state transitions than controls. These findings add to the growing literature on neural sex differences and may be related to the sex difference in focal versus diffuse attention.

## Introduction

Attention-Deficit/Hyperactivity Disorder (ADHD) affects at least 5% of children and adolescents [1–4]. Its primary symptoms include inattention, impulsivity, and hyperactivity, with associated deficits in self-regulation, executive function, and working memory [1, 5]. Children diagnosed with ADHD often continue to experience these symptoms into adulthood, with about one in seven continuing to meet full diagnostic criteria as adults [6–7]. The practical consequences of ADHD are reflected in social and academic difficulties for children [8–10], as well as social and employment issues for adults [11].

There are two- to three boys for every girl diagnosed with ADHD [12], as well as sex differences in how the symptoms manifest. For instance, boys with ADHD show more impulsive behaviors and larger deficits in executive function, achievement, and sociality, whereas girls with ADHD on average have lower intelligence and exhibit more internalizing (e.g., depression) symptoms and fewer disruptive behaviors [13–15]. Neuroimaging studies, including magnetic resonance imaging (MRI) have revealed both structural and functional differences between children with ADHD and their typically developing peers, as well as sex differences among children with and without ADHD.

Here, we first review the neuroimaging literature on both ADHD and sex differences, (also see [16–17]), and then describe how we utilized resting-state functional MRI (or rs-fMRI) to investigate sex differences in brain network dynamics in children with ADHD and their typically developing peers. Resting-state functional MRI is often used to assess the temporal coherence (correlation) of activity between multiple brain regions, and we extend this approach by using Hidden Markov Modeling (HMM) to identify latent state changes across sex and for children with and without an ADHD diagnosis.

### Prior neuroimaging studies

Neurological deficits have long been thought to contribute to ADHD [2], and the results from brain imaging studies are consistent with this view. In terms of brain structure, children with ADHD have been found on average to have a smaller caudate [18], cerebellum [19–20], prefrontal cortex [19], and overall white matter volumes [21] compared to their typically developing peers. Aberrant functional connectivity has also been found in children with ADHD, including greater connectivity among multiple brain regions. These include the cerebellum, left fusiform, right inferior temporal gyrus, left supplementary motor cortex [22], as well as areas in the prefrontal cortex (PFC) and the default mode network (DMN) [23]. At the same time, there appear to be other regions with lower levels of connectivity in children with ADHD relative to other children, including the superior parietal cortex [24], left precuneus [21, 24], and thalamus [21], as well as overall reductions in temporal coherence.

In typically developing children, temporal coherence generally increases with age, and the relatively poor temporal coherence in children with ADHD might reflect a developmental delay. Either way, decreased temporal coherence between the DMN and other cortical regions is often found for children with ADHD [25–28]. Indeed, abnormal DMN functional connectivity is a frequently studied aspect of ADHD [25, 27–28].

Moreover, children with ADHD often differ from other children in terms of functional connectivity involving regions of the prefrontal cortex. In a resting-state study, Bos et al. found increased but more diffuse connectivity patterns in the medial PFC and the inferior frontal gyrus in children with ADHD relative to typically developing children [29]. Other studies have reported similar levels of hyperfrontality [30–31], but still others have indicated hypofrontality [32–36]. Lower connectivity between the precuneus and ventromedial PFC has also been identified in adults with ADHD [37–39], as well as other general decreases in prefrontal activity with children with ADHD [22], and in the ventrolateral [40] and lateral [21] PFC.

These neuroimaging findings are consistent with differences in multiple brain networks of children with ADHD relative to their typically developing peers. Differences in the DMN suggest that children with ADHD may not engage in the same forms of mind wandering as typically developing children, and differences in the frontal-striatal, frontoparietal control, and attention (dorsal and ventral) networks are consistent with deficits in executive functioning and cognitive control. The relation between the DMN and attentional networks is also important: specifically, for shifting from self-referential cognition associated with episodic memory to the attentional focus needed to address specific, non-self-referential demands [41]. In other words, many children with ADHD likely have deficits in ease of shifting between networks that might be of functional importance [28, 42]. A recent study used whole-brain functional connectivity to find a neuromarker of sustained attention [43]. This neuromarker was able to successfully predict measures of sustained attention (symptom of ADHD), showing that whole-brain resting-state network activity holds informational content related to measures of attention, potentially relating to subjects with ADHD as well.

It is currently unclear whether the sex difference in the prevalence of ADHD is an exaggeration of more general sex differences in verbal, motor, or brain function or reflects something unique to ADHD. Generally, many sex differences are similar to those found in ADHD. For instance, sex differences have been found in functional connectivity within both the DMN and the sensorimotor cortices [44–45]. Apart from average differences in activation or connectivity, men tend to be more variable in many cognitive and behavioral domains [46], and more variable in neural patterns. As a potentially relevant example, Johnson and Bouchard found a large sex difference (*d* = .9) on a composite measure of diffuse versus focused attention, after controlling for general intelligence [47]. Men on average were better able to maintain attentional focus, but were also more variable than women as a group. Whether this would manifest as greater variability in boys than girls in switching between networks among typically developing children or children with ADHD is unknown and merits investigation.

### Current study

Although many potentially important ADHD-related and sex differences in brain structure and functions have been identified, relatively little is known about the temporal dynamic changes of brain activity in these individuals. In one study, Alba et al. observed no significant group differences in averaged connectivity between subjects with ADHD and controls, although ADHD subjects showed more variability in connectivity over time in frontal and parieto-occipital regions relative to controls [48]. However, to our knowledge, no other study has modeled dynamic changes without averaging over time in *f*MRI, as is commonly done in static functional connectivity analyses. Given sex differences in ADHD symptomatology, the over-representation of males among individuals with ADHD, and the sex difference in focused-diffuse attention, the study of sex and diagnostic differences in temporal dynamics of state, or network, changes in resting-state *f*MRI could provide unique insights into ADHD and sex differences linked to ADHD more specifically. Here, we use computational modeling to estimate latent, or hidden, brain states and to quantify whole-brain changes in state dynamics as a function of ADHD diagnosis and sex.

## Results

Subject characteristics including sample size, age, and basis for ADHD diagnosis can be found in Table 1. Age was initially utilized as a covariate in the main analyses, but the independence assumption between the covariate and treatment effects was violated, rendering the use of age as a covariate inappropriate. Therefore, the presented analyses do not include age as a covariate. When age was included, the main conclusions did not change, and details of this analysis can be found in the supplementary materials.

**Table 1:** Subject characteristics.

				ADHD*		
Sex	Diagnosis	N	Age	Hyperactive/Impulsive	Inattentive	Combined
Girl	ADHD	99	10.64 (2.71)	3.70%	46.30%	50.00%
Boy	ADHD	347	11.92 (2.98)	4.02%	37.95%	58.04%
Girl	Control	251	11.39 (2.98)	-	-	-
Boy	Control	257	11.81 (2.77)	-	-	-

N = number of analyzed scans.

*Percentage of each type of diagnosis is shown for participants with ADHD. The combined diagnosis lists children showing both hyperactive/impulsive as well as inattentive behaviors.

**Fig 1:**
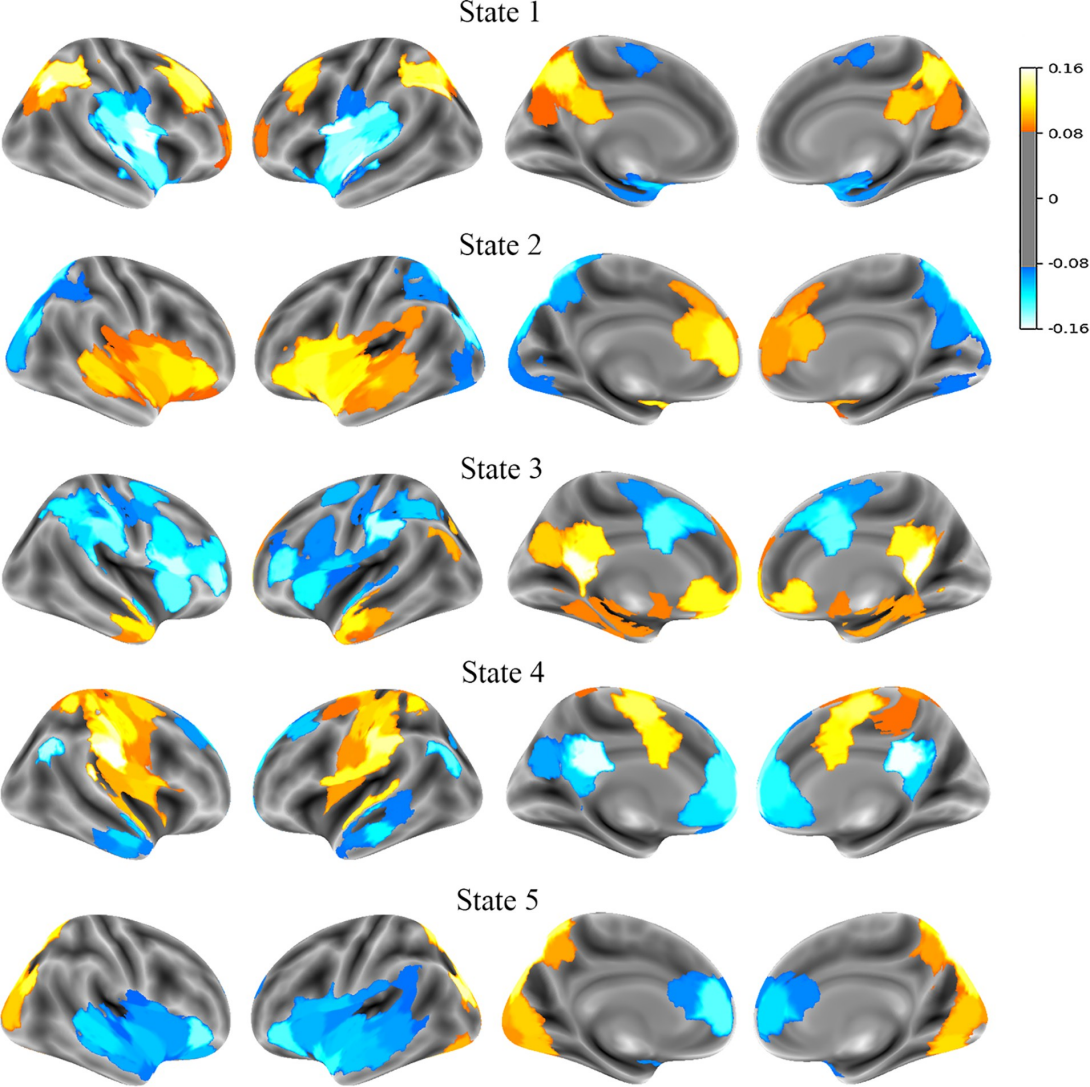
Latent state mean activation maps. From left to right, mean activation maps for each of the five latent states are shown from the right lateral, left lateral, left medial, and right medial positions.

Latent states from the rs-fMRI time series data were estimated using Hidden Markov Modeling (HMM) for each group separately. To resolve potential differences in the nature of each state for the four groups (e.g., one latent state for girls with ADHD might differ from another latent state for boy controls), and to combine similar states across the groups, k-means clustering was performed. A five-cluster model was chosen, using the mean activation maps for each of the 20 estimated states (i.e. five states for each of the four groups). Results from the cluster analysis showed that each cluster appropriately contained only one mean activation map from each experimental group (sex × diagnosis). In other words, the HMM identified five states that were similar across the four groups. Thus, mean activation maps were calculated for each of the five states by averaging maps across all four groups. These were then transformed into brain space and are plotted in Fig 1. Un-averaged maps for each of the four groups are listed in the supplementary materials, and show minimal variation from the averaged maps. The notion of latent states is loosely similar to networks derived from intrinsic functional connectivity. However, characterizations and estimations between HMM and other clustering algorithms of functional connectivity are qualitatively different. That being said, the five latent states are similar to aspects of the 1) dorsal attention (DAN) and frontoparietal control networks (FPC), 2) ventral attention network (VAN), 3) default mode network (DMN), 4) somatomotor network (SMN), and 5) visual network (VIS), respectively.

**Fig 2:**
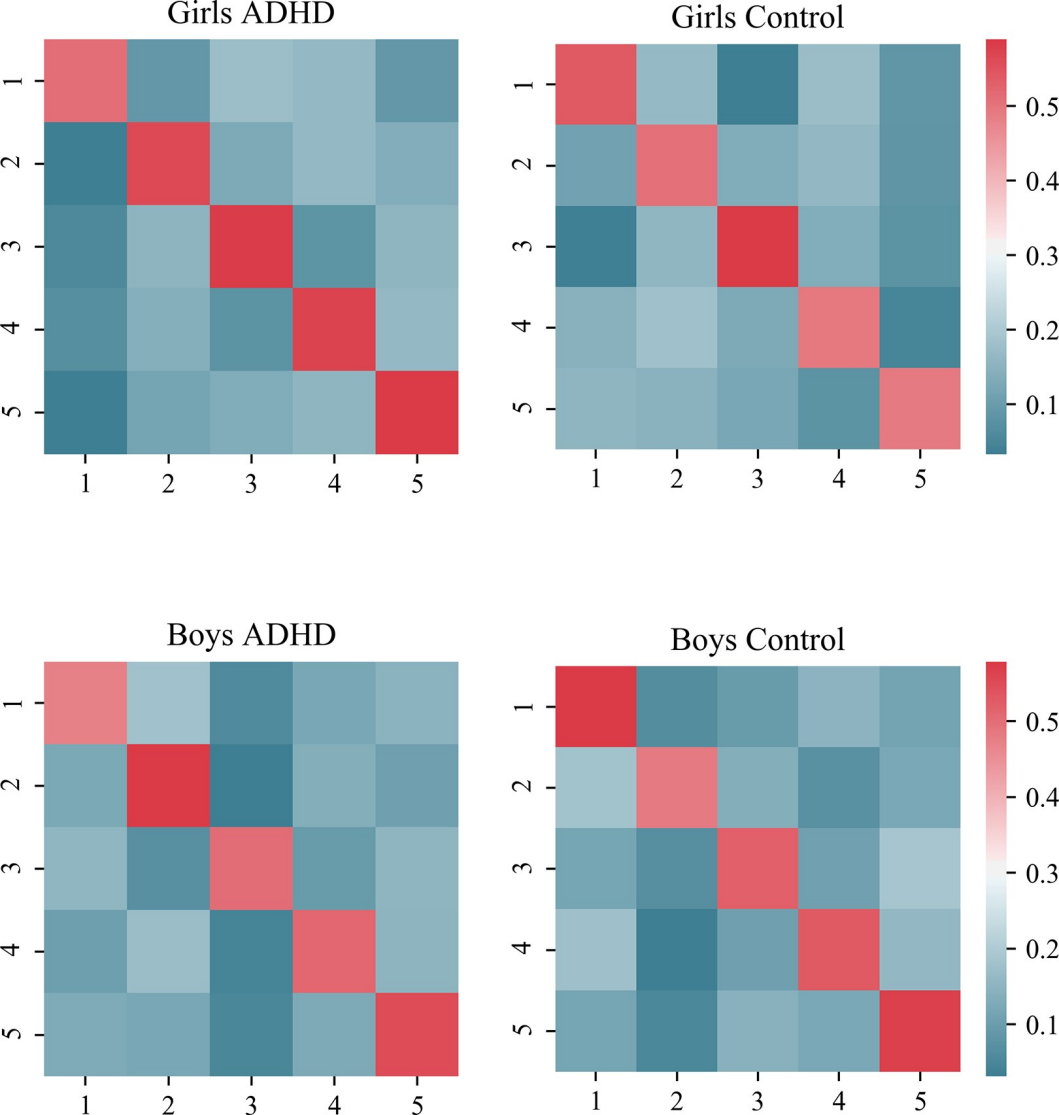
Transition probability matrix (TPM) heat maps. TPMs are shown for each group. Each cell lists the estimated probability of switching from one particular state (rows), to another state (columns).

Next, fractional occupancy was calculated separately for each group, which provided the overall proportion of time each state was visited throughout the time series. These estimates averaged across subjects can be found in Table 2. Girls and boys with and without ADHD appeared to spend the majority of their time in different states. This was confirmed by a significant three-way interaction in FO estimates (sex × diagnosis × state), *F*(4, 2460) = 666.32, *p* < .001, *η*_*p*_^*2*^ = .52. In particular, typically developing boys and girls had the highest fractional occupancy in the fourth latent state, whereas fractional occupancy for girls and boys with ADHD was the highest in states five and three, respectively. Transition probability matrices (TPMs) were estimated for each group to detail the probability of switching from one particular state to another. As is apparent in Fig 2, the TPMs for every group show a general tendency to stay within the same state (On-diagonal: *M* = .53, *SD* = .04) compared to switching to other specific states (Off-diagonal: *M* = .12, *SD* = .04). Apart from a higher tendency to remain in the same state as the preceding time point, transition probabilities appear to be nonspecific, such that there are similar probabilities for switches across states (i.e. similar Off-diagonal values). While there are subtle asymmetries in transition directionality (i.e. more likely to switch from state 1 to 3 than 3 to 1 in girls with ADHD, yet the opposite pattern in boys with ADHD), no consistent pattern is evident that would explain overall differences between sex or diagnosis. State switching differences are thus more prevalent in terms of their general duration and frequency.

**Table 2:** Fractional occupancy.

	Girl		Boy	
State	ADHD	Control	ADHD	Control
1	.08	.24	.24	.09
2	.24	.16	.17	.17
3	.16	.20	.32	.21
4	.19	.30	.07	.29
5	.32	.10	.20	.24

Fractional occupancy, the proportion of time spent in each state are shown for each experimental group and for all states, such that each column sums to one.

### Inter-transition interval (ITI)

**Fig 3:**
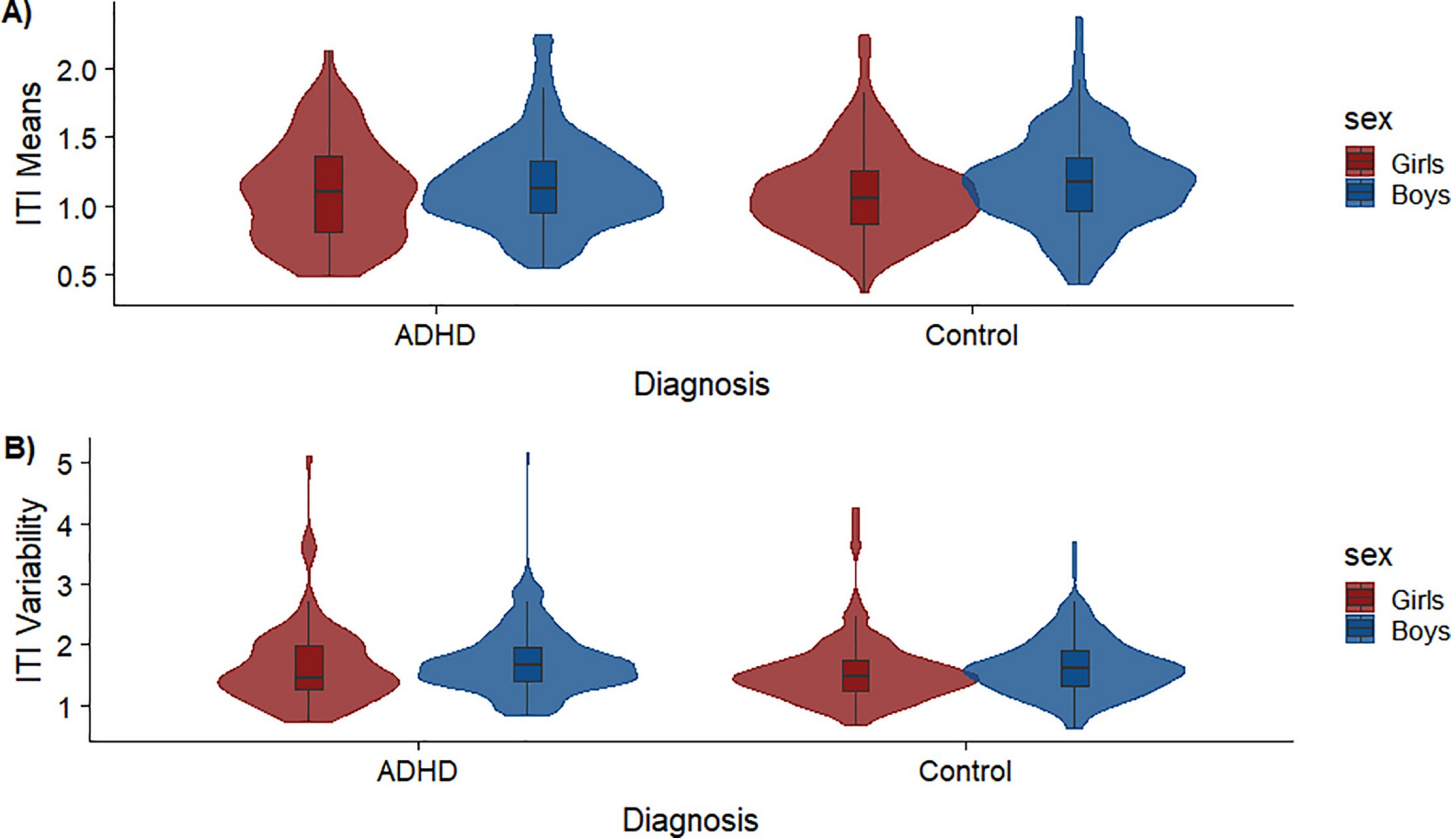
Inter-transition intervals (ITI) by group. Violin plots showing A) mean estimates and B) variability estimates for the ITIs, a measure depicting how long subjects dwell in a particular state before switching to another. ITI is plotted on the x-axis by Diagnosis, grouped by females (pink) and males (blue).

Fig 3 displays the group means and variability for the inter-transition interval (ITI), which reflects the number of consecutive time points associated with a particular state before switching to a new state. Across the whole data set, the measures for eleven resting-state runs were identified as outliers (three absolute standard deviations of the scaled data [49]) and were thus removed from subsequent testing. A two-way between-subjects ANOVA revealed that boys (*M* = 1.17, *SD* = 0.33) dwelled longer on average than girls (*M* = 1.10, *SD* = 0.33) in any given state, *F*(1, 939) = 8.63, *p* = .003, *η*_*p*_^*2*^ = .01. There were no differences for mean ITI estimates across diagnostic category; children with ADHD (*M* = 1.15, *SD* = 0.34), control children (*M* = 1.14, *SD* = 0.33), *F*(1, 939) < 0.01, *p* = .95, *η*_*p*_^*2*^ < .001. Additionally, there was no interaction between sex and diagnostic category, *F*(1, 939) = 0.02, *p* = .90, *η*_*p*_^*2*^ < .001.

To further explore which states were driving sex differences, ITI’s for each individual state were similarly calculated as the average number of consecutive time points in each state before a switch to a new state. As can be seen in the left side of Table 3, boys dwelled longer than girls in the majority of states, with the exception of state five, where the trend in means was reversed (Girls > Boys; *p* > .05). This pattern of longer dwelling times was primarily driven by states two, three, and four (*p*’s < .001, average *d* of 0.31). These relate to networks similar to the VAN, DMN, and SMN, respectively.

**Table 3:** State specific ITI means and variability.

	ITI Averages			ITI Variability		
State	Girls *M*(*SD*)	Boys *M*(*SD*)	*p* value (*d*)	ADHD *M*(*SD*)	Controls *M*(*SD*)	*p* value (*d*)
1	1.16 (0.46)	1.17 (0.52)	.482 (0.02)	1.40 (0.55)	1.45 (0.51)	.054 (0.09)
2	1.08 (0.41)	1.23 (0.51)	**< .001 (0.31)**	1.48 (0.53)	1.43 (0.53)	.545 (0.10)
3	1.04 (0.59)	1.21 (0.55)	**< .001 (0.31)**	1.45 (0.60)	1.29 (0.54)	**.002 (0.28)**
4	0.96 (0.52)	1.15 (0.54)	**< .001 (0.34)**	1.33 (0.60)	1.34 (0.52)	.100 (0.01)
5	1.03 (0.61)	0.92 (0.64)	.052 (0.18)	1.07 (0.64)	1.17 (0.62)	.055 (0.15)

Sex differences in ITI averages (left), and diagnosis differences in ITI variability (right). Means, standard deviations, *p* values, and Cohen’s *d* values are presented for each state.

The standard deviation of ITI was used as an indicator of variability. Children with ADHD were more variable as a group (*M* = 1.70, *SD* = 0.55) than control children (*M* = 1.60, *SD* = 0.52), *F*(1, 939) = 4.69, *p* = .03, *η*_*p*_^*2*^ = .005. There was slightly greater variability in ITI among boys (*M* = 1.68, *SD* = 0.50) than girls (*M* = 1.59, *SD* = 0.59) in state switching, albeit the difference was not statistically significant, *F*(1, 939) = 3.32, *p* = .07, *η*_*p*_^*2*^ = .004. The interaction between sex and diagnosis was also not significant, *F*(1, 939) = 0.001, *p* = .98, *η*_*p*_^*2*^ < .001. Analogous to ITI means, to further explore which individual states were driving diagnosis differences in ITI variability, state-specific estimates were calculated. As can be seen on the right side of Table 3, differences in ITI variability were mainly driven by higher variability in ADHD than control children in state three (DMN; *p* = .002, *d* = 0.28).

### Number of transitions (NT)

**Fig 4:**
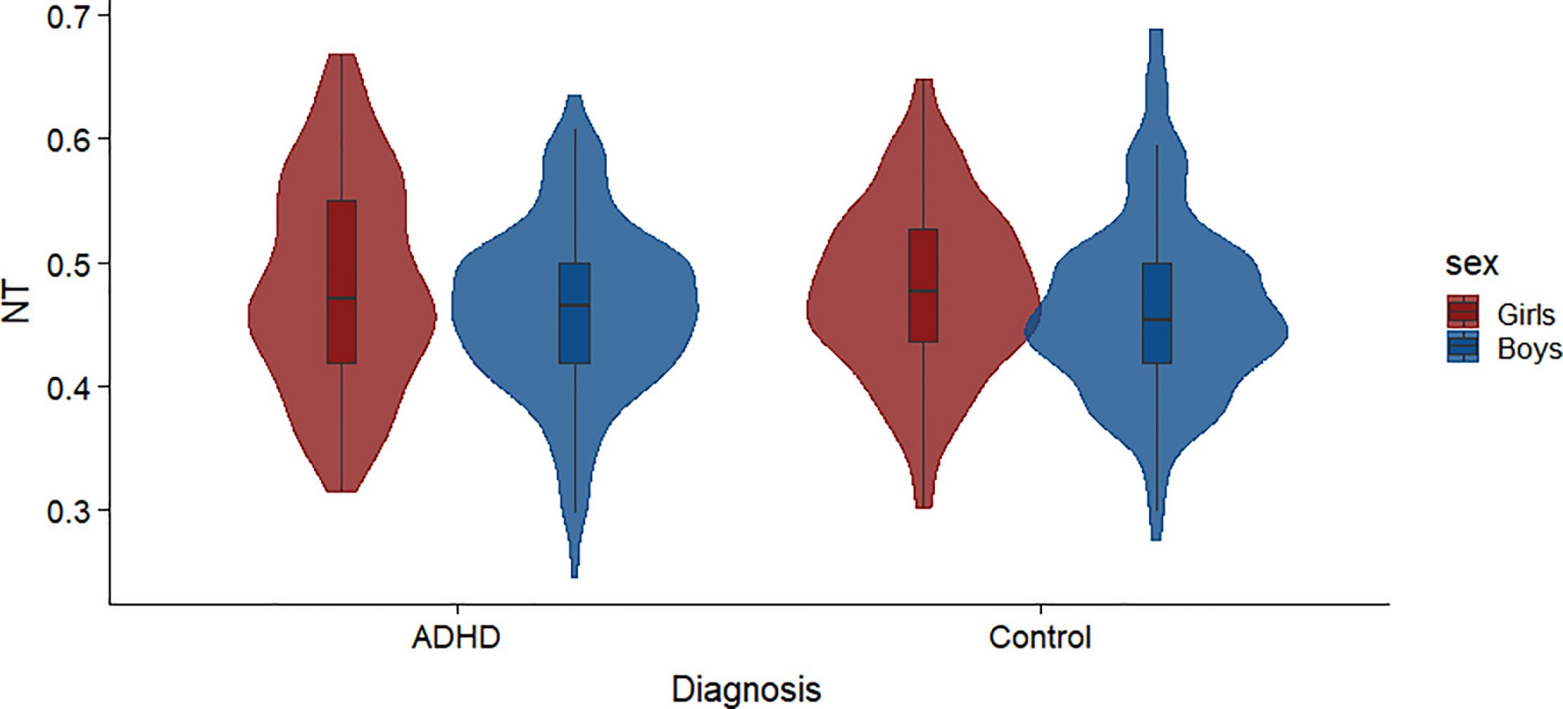
Number of transitions (NT) by group. Violin plot showing the NTs, a measure of how often subjects switch from one state to another across the time series. NT is plotted on the x-axis by Diagnosis, grouped by females (pink) and males (blue).

The number of transitions (NTs) indexes the frequency of state changes across the time series, divided by the overall length of the time series for each subject. Data were again screened for outliers, and eight runs were identified and removed from the analysis. Fig 4 shows the group means and variability for NT. A between-subjects ANOVA revealed that girls (*M* = 0.48, *SD* = 0.07) had more NTs than boys (*M* = 0.46, *SD* = 0.07), *F*(1, 942) = 11.25, *p* = .001, *η*_*p*_^*2*^ = .01. This result is consistent with the numerical sex difference in ITI, which was to be expected, as the ITI and NT measures are highly correlated (*r* = -.94, *p* < .001). Neither the main effect of diagnosis, *F*(1, 942) = 0.06, *p* = .80, nor the interaction between diagnosis and sex, *F*(1, 942) = 0.18, *p* = .67, were significant.

## Conclusions

There is now ample evidence for structural and functional connectivity differences across the sexes and for children with ADHD relative to their typically developing peers. Studies have shown functional connectivity differences, where women exhibited more coherent connectivity in regions overlapping with the DMN, and men generally having stronger connectivity in sensorimotor areas [45]. In a similar study, whole-brain functional connectivity was used to successfully predict participant sex using multivariate pattern classification methods [50]. In line with the findings of Ritchie et al. [45], some of the most important features that were predictive of participant sex were functional connections in the DMN as well as SMN areas.

Neuroimaging studies of children with ADHD show structural and functional differences compared to their typically-developing peers. Atypical functional connectivity in ADHD has been identified in prefrontal, striatal, cerebellar, and temporal regions, along with regions commonly found in the DMN [21–24]. The majority of the associated studies have examined static connectivity (averaged over time), and we moved one step beyond this with the use of HMM to examine temporal dynamic changes of brain activity and to identify latent states using resting-state *f*MRI. While HMM characterizes data as a series of latent states, the use of HMMs assume that the system under scrutiny is in one state at any given point in time (states are mutually exclusive). While the inferred state at time *t* is the reflection of the highest probability over other potential states, it could be the case that two states at time *t* have relatively similar probabilities. Thus, there can be some uncertainty in the model at equivocal time points as to which states are truly reflected in neural data.

### Sex differences

Our results contribute to this growing body of research on sex differences in the brain. Along with differences in static functional connectivity [45], we revealed dynamic state changes between boys and girls with and without an ADHD diagnosis. The two metrics of state switching (ITI and NT) suggested that boys dwell longer than girls in a given state before switching to a new one, independent of whether or not they were diagnosed with ADHD. The functional implications are not clear at this time but one possibility is that boys do not switch between functional networks as fluidly as girls, and may especially involve dwelling in states relating to the VAN, DMN, and SMN.

We cannot be certain because Johnson and Bouchard [47] assessed adults, but our findings are consistent with more focused attention in males and diffuse attention in females. As noted, the men in their study were also more variable as a group, indicating that many men were able to maintain task-specific attention (during psychometric testing) but others had frequent lapses in attention. A similar pattern of greater variability in ITI and NT (Figs 3 and 4) emerged for our control children, but the differences were not statistically significant, perhaps because resting-state conditions do not require focused attention. While consistent with previous research, whether the observed differences in brain activity are innate or reflect societal gender roles remain empirically unanswered, an important note that merits further investigation.

In any case, sex differences in attentional focus might result from slow switching to or from attention networks to frontoparietal control and/or DMN networks. Dwelling in the DAN or VAN would facilitate performance in attention-demanding tasks, whereas dwelling in another state and delaying the switch to attention networks would hamper performance. One result would be greater variability among males. State switching deficits might also contribute to some of the impulsivity and executive function symptoms commonly seen in boys with ADHD. However, many of the control boys showed the same pattern and thus ITI and NT did not interact with diagnosis. Again, the resting-state condition, with few if any external distractors, might have obscured any underlying differences.

### ADHD

No sex differences were observed in the variability of state switching. However, children (both boys and girls) with ADHD were found to be more variable in state switching than were typically developing controls. The children with ADHD also tended to dwell in different states than typically developing children. The functional significance of dwelling in one state or another cannot be determined at this time, but suggests that even during rest the patterns of global brain activity and change in the distribution of this activity differ in children with ADHD relative to typically developing children. This result adds to the vast literature detailing aberrant static functional connectivity in ADHD. Along with general connectivity strength differences, we found that typical dynamic state switching may be altered in children with ADHD, showing greater variability than controls that in turn might be specific to regions within the DMN. Furthering research in areas related to the classification of various neurological and psychiatric disorders should begin to address the degree of non-stationarity in neural signals that may be dissociable at the individual level. By using dynamic modeling approaches such as HMM, we can begin to study the structure of intraindividual variation in cases where latent categorical processes may be present [51]. Many neural and psychological behaviors occur in real time at the level of individuals [52].

Further research that continually utilizes larger samples will be beneficial. The sample in the current analysis was asymmetric (smallest sample in girls with ADHD). However, in a sub-sample analysis, where subjects were randomly sampled from the other three groups to match the size of the smallest group, results remained unchanged. For ITI, the main effect of sex was still present, *F*(1, 388) = 9.09, *p* = .003, *η*_*p*_^*2*^ = .02, as well as the main effect of diagnosis in ITI variability estimates, *F*(1, 388) = 6.79, *p* = .01, *η*_*p*_^*2*^ = .02. For NT, the main effect of sex also remained unchanged, *F*(1, 388) = 4.31, *p* = .04, *η*_*p*_^*2*^ = .01. More details as well as visualizations from the sub-sample analysis can be found in the supplementary materials. Another important consideration is that whereas concatenating data from multiple individuals (as in the current analysis) may not fully reveal individual-specific dynamic changes, analysis based on intraindividual variation seems like an appealing future direction.

Developmental studies tracking changes in behavior and neural function across the lifespan may also elucidate dynamic shifts in activity as a function of time. Along with resting-state *f*MRI, experiments using task-based paradigms can also show dynamic changes in response to various stimuli (i.e. attention tasks). The current study elucidated sex and ADHD differences in neural state switching, and while we may hypothesize how this might relate to behavioral deficits between males and females with and without ADHD, directly relating this to behavioral performance in future research seems crucial. Overall, we found that resting-state *f*MRI carries dynamic information pertaining to subject sex and ADHD diagnosis.

## Materials and methods

### Subjects

Data from subjects recruited for the ADHD-200 consortium (http://fcon_1000.projects.nitrc.org/indi/adhd200/) were selected for the current study, as shown in Table 1. The consortium involved multiple sites that tested both ADHD and typically developing control subjects. The sites were the Kennedy Krieger Institute, New York University Child Study Center, Peking University, Oregon Health and Science University, and the NeuroIMAGE sample. Fifty-four girls with ADHD (160 controls), and 225 boys with ADHD (179 controls) were included in the current study. As many subjects had multiple resting-state scans, this resulted in 99 scans for girls with ADHD (251 scans for controls) and 347 scans for boys with ADHD (257 for controls).

### MRI acquisition and preprocessing

Across the multiple sites, different models of scanners (all at 3T strength) and scanning parameters were employed. See [53] for full details, as well as http://fcon_1000.projects.nitrc.org/indi/adhd200/. Some of the notable differences in data acquisition included the total scan duration (from three minutes and 32 seconds to eight minutes and 47 seconds), repetition time (TR; from 1960 to 2500 ms), and voxel size (3-3.8 mm in-plane resolution and 3-4 mm slice thickness).

Publicly available versions of the pre-processed imaging data were used for the current study [53]. Preprocessing was implemented by the NeuroBureau on the Athena computer cluster at Virginia Tech’s Advanced Research Computing center (http://www.arc.vt.edu). The preprocessing pipeline, also known as the ‘Athena processing pipeline’, was based on a combination of functions from the AFNI [54] and FSL [55] toolboxes. The pipeline is briefly described here for convenience. The structural images were skull-stripped, segmented into cerebro-spinal fluid (CSF), white matter (WM), and gray matter (GM) probability maps, non-linearly warped to an age-specific MNI template [56], resampled to 1-mm isotropic voxels, and blurred using a 6-mm FWHM Gaussian filter. Preprocessing of the rs-fMRI included discarding the first four volumes of each run, slice-timing correction, realignment to the first volume, and linear co-registration with the structural data. Warping parameters determined for the structural data were then applied to the functional data and the images were resampled into 4-mm isotropic voxels. Mean WM and CSF time courses were included along with the six motion parameters and a third-order polynomial as nuisance regressors in a GLM. The resulting time series were then band-pass filtered (0.009 – 0.08 Hz) [44, 57] and spatially smoothed with a 6-mm FWHM Gaussian filter. Time courses for each subject were then extracted from the processed functional data using the CC-200 brain parcellation scheme [53, 58].

### Data analysis

Data analysis was performed using Python v. 3.6.4 and R v. 3.5.0. Code and supplementary materials can be found online at https://osf.io/xqewf/. Analysis of the rs-fMRI data first employed Bayesian Hidden Markov Modelling (HMM). The method was recently adapted for use with rs-fMRI to assess how network-related activity exhibits stability versus change across time [59–60]. In-depth descriptions of HMM can be found elsewhere [59, 61–63]. For our purposes, it is used to describe time series measurements as a series of hidden, or latent, model states that correspond to networks of functionally-connected brain regions. HMM has been shown to be superior to alternative techniques of modelling dynamic connectivity, such as the sliding window approach, [64], as these alternatives may lead to unstable results when scan times are relatively short (for further discussion, see [59, 65]). While advantageous in various ways, HMM also may share the limitations of these other methods, depending on the quality and amount of data available in terms of true state estimations and ensuing metric characterizations.

Our analysis of HMM parameter estimates was based on the work from Hutchison and Morton [66]. A model consisting of five such states was used for the current analyses. To choose the optimal number of network states, the following steps were performed. First, the time series data were individually z-scored and separately concatenated across subjects in their respective group (ADHD/control × boy/girl) [67]. Next, the resulting time series were subjected to multiple HMMs, with the number of states ranging from 1 to 10. Finally, when all models were fitted, the log probability scores for each were compared visually based on diminishing improvements in fit with additional states (i.e., the elbow method). These comparisons suggested that for each of the four groups, five states captured most of time-related variation. Thus, these analyses suggested a five-state model was optimal for all groups (see supplementary materials).

After the final HMM was estimated for each group, the latent state properties for each group were inspected in three ways. First, mean activation maps for each latent state were extracted and plotted as whole-brain maps. Second, *transition probability matrices* (TPMs) were extracted from the model, which for each group details the probability of switching from one particular state to another. Third, *fractional occupancy* (FO) which is the proportion of all time points (TRs) that correspond to a particular state, and which indicates the states that were most prevalent across subjects, was calculated and inspected for each group. A note to consider is that FO values might be minimally influenced by inter-group differences in mean activation maps for various states. While the majority of variance agrees across groups for each state, there are subtle differences in the spatial extent between groups. Thus, differences between groups in FO values might be partly attributable to these differences. However, in the absence of any clear pattern distinguishing between sex or ADHD diagnosis, differences in FO values should be interpreted with caution.

Additionally, two critical measures were used in analyses to estimate state change dynamics. *Inter-transition interval* (ITI) is the number of consecutive time points that subjects dwell in any one state before switching to another, and was averaged across all states for each individual subject. Finally, the *number of transitions* (NT) was calculated as the number of times a change between any two states was detected. This measure was averaged across the number of time points in each individual time series and thus indicates the frequency of state switching. Using these measures, the primary analyses focused on changes in mean and standard deviation estimates for the inter-transition interval, as well as the average number of transitions across the four groups of subjects (Girl ADHD, Girl control, Boy ADHD, and Boy control).
